# The effects of hip muscle strengthening on knee load, pain, and function in people with knee osteoarthritis: a protocol for a randomised, single-blind controlled trial

**DOI:** 10.1186/1471-2474-8-121

**Published:** 2007-12-07

**Authors:** Kim L Bennell, Michael A Hunt, Tim V Wrigley, David J Hunter, Rana S Hinman

**Affiliations:** 1 Centre for Health, Exercise & Sports Medicine, School of Physiotherapy, University of Melbourne, Australia; 2 New England Baptist Hospital, Boston, MA, USA

## Abstract

**Background:**

Lower limb strengthening exercises are an important component of the treatment for knee osteoarthritis (OA). Strengthening the hip abductor and adductor muscles may influence joint loading and/or OA-related symptoms, but no study has evaluated these hypotheses directly. The aim of this randomised, single-blind controlled trial is to determine whether hip abductor and adductor muscle strengthening can reduce knee load and improve pain and physical function in people with medial compartment knee OA.

**Methods/Design:**

88 participants with painful, radiographically confirmed medial compartment knee OA and varus alignment will be recruited from the community and randomly allocated to a hip strengthening or control group using concealed allocation stratified by disease severity. The hip strengthening group will perform 6 exercises to strengthen the hip abductor and adductor muscles at home 5 times per week for 12 weeks. They will consult with a physiotherapist on 7 occasions to be taught the exercises and progress exercise resistance. The control group will be requested to continue with their usual care. Blinded follow up assessment will be conducted at 12 weeks after randomisation. The primary outcome measure is the change in the peak external knee adduction moment measured during walking. Questionnaires will assess changes in pain and physical function as well as overall perceived rating of change. An intention-to-treat analysis will be performed using linear regression modelling and adjusting for baseline outcome values and other demographic characteristics.

**Discussion:**

Results from this trial will contribute to the evidence regarding the effect of hip strengthening on knee loads and symptoms in people with medial compartment knee OA. If shown to reduce the knee adduction moment, hip strengthening has the potential to slow disease progression.

**Trial Registration:**

Australia New Zealand Clinical Trials Registry ACTR12607000001493

## Background

Osteoarthritis (OA) is a chronic, localised joint disease affecting approximately one-third of adults, with the disease prevalence increasing with advancing age [1]. Concomitant with this high prevalence is a large economic cost, with direct and indirect costs estimated to be $23.9 billion in Australia in 2007 [2]. Indeed, given the changing demographics of the adult population [3], expectations are for the prevalence of disease and its burden on the health care system to increase in coming decades [4]

The knee is the most common weightbearing joint affected by OA, with the disease predominantly affecting the medial compartment of the tibiofemoral joint [5,6]. Patients with knee OA frequently report symptoms of knee pain and stiffness as well as difficulty with activities of daily living such as walking, stair-climbing and housekeeping [7]. Ultimately, pain and disability associated with the disease lead to a loss of functional independence and a profound reduction in quality-of-life.

To date, most knee OA research examining treatment for knee OA has focused on surgical or pharmacological strategies. Although effective, these types of interventions have many potential side effects and are expensive [8]. Thus, recent knee OA clinical guidelines reinforce the importance of non-pharmacological strategies in the management of the condition [9,10]. However, there is an absence of high quality evidence to support the use of such therapies [9]. Given that surgical replacement of the knee is the only recognized treatment for end-stage structural degeneration of the joint, there is an urgent need to develop new treatment interventions capable of effectively reducing the personal and societal burden of knee OA.

Increased loading across the joint has been implicated in the progression of knee OA severity [11]. The role of gait analysis in the quantification of dynamic joint load has received much attention in the literature in light of the difficulty in performing *in vivo *measurement of joint loading during movement [12-14]. From this research, the external knee adduction moment, an indirect measure of load in the medial compartment of the tibiofemoral joint [15], has emerged as an important and widely accepted biomechanical marker of knee load.

Cross-sectional studies demonstrate that patients with knee OA have a higher peak knee adduction moment during walking when compared to healthy age-matched controls [16,17]. It is also likely that the higher prevalence of medial compared with lateral tibiofemoral joint OA is the result of differences in the relative loading within the tibiofemoral joint. The external knee adduction moment determines load distribution across the medial and lateral tibial plateaus [18-20], with force across the medial compartment almost 2.5 times that of the lateral [15]. It has also been reported that for patients with knee OA, the magnitude of the adduction moment is predictive of clinical outcomes such as severity of knee pain [21] and radiographic disease [22]. Lastly, longitudinal studies have demonstrated that as little as a one-unit increase in the adduction moment is associated with up to a 6.5-fold increase in the risk of disease progression [11]. Given the importance of the knee adduction moment with regard to both symptom severity and disease progression in knee OA, conservative strategies to reduce the knee adduction moment constitute a logical rehabilitative approach.

A variety of exercise programs for knee OA have been described in the literature. These have included general aerobic exercise programs such as walking or cycling as well as more specific programs involving strengthening of particular muscle groups and/or flexibility exercises. Studies investigating the effects of strengthening in patients with knee OA have generally focussed on improving quadriceps strength [23-25]. However, little attention has been paid to improving the strength of other lower limb muscle groups such as the hip abductors and adductors. Due to the lack of research investigating their efficacy, such exercises are not routinely prescribed for knee OA.

Recent evidence, however, suggests that hip abductor and adductor muscle strength may be important for reducing the knee adduction moment [26-28]. During walking, these muscles stabilise the pelvis on the hip joint in the frontal plane. The position of the pelvis can alter the position of the body's centre of mass and thereby alter loads at the knee joint. It is postulated that during single leg stance support in gait, weakness of the ipsilateral hip abductors may drop the pelvis toward the contralateral swing leg which can shift the body's centre of mass away from the knee joint centre and increase the knee adduction moment. Importantly, a recent longitudinal cohort study found that people with a lower external *hip *adduction moment (suggesting weaker hip abductor muscles) demonstrated more rapid knee OA progression [26].

Less is known about the hip adductor muscles in relation to knee OA but they may also help reduce the knee adduction moment, particularly in a varus malaligned knee. By virtue of their attachment to the distal medial femoral condyle, the adductors could eccentrically restrain the tendency of the femur to move further into varus. Yamada *et al.*[29] found that patients with knee OA demonstrated stronger hip adductors compared with age-matched controls, and that those with more severe OA had even stronger adductors than their less severe counterparts. They hypothesised that this increased strength may be due to greater use of the hip adductors in an attempt to lower the knee adduction moment.

Given that hip muscle strength has the potential to alter knee load, we hypothesise that hip muscle strengthening may be a novel intervention for rehabilitation of knee OA patients that slows disease progression and reduces symptoms. However, this has not been evaluated to date. The primary aim of this trial is to determine whether strengthening of the hip muscles in people with medial compartment knee OA reduces knee joint loading as quantified by the peak external knee adduction moment. The secondary aim is to determine whether hip strengthening can reduce knee pain and improve function. It is hypothesised that a 12-week program of strengthening the hip abductor and adductor muscles will i) reduce the knee adduction moment; and ii) improve pain and physical function.

## Methods/Design

### Design

**Figure 1:**
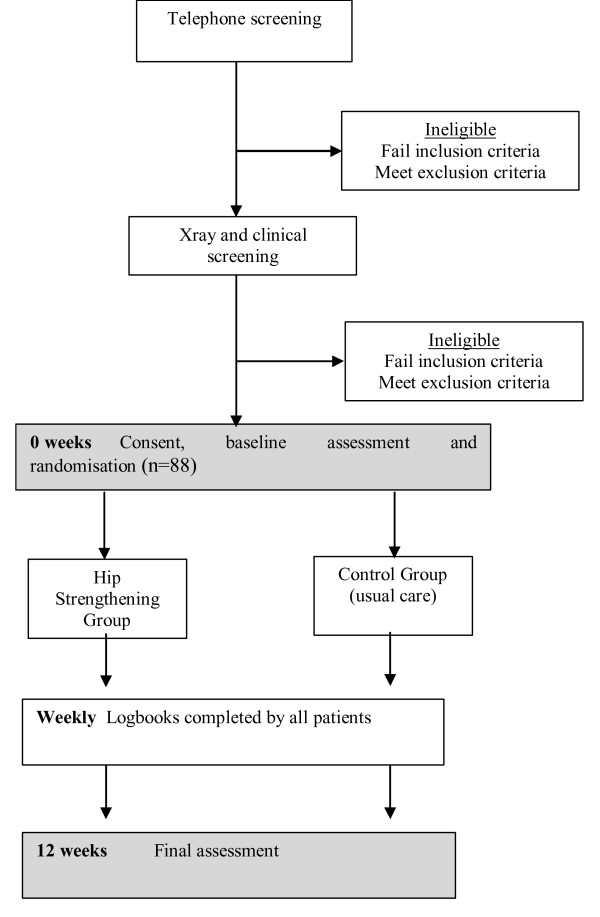
Trial protocol.

This will be a single-blind randomised controlled trial (Figure 1). Potential participants will undergo telephone screening followed by clinical examination by a physiotherapist to ensure eligibility. A screening x-ray will be performed to quantify knee joint alignment and assess disease severity. Eligible participants will then be stratified by disease severity (Kellgren and Lawrence grade 2, 3 and 4) [30] and randomly allocated in permuted blocks of 4 to 6 to hip strengthening or control groups. The randomisation sequence will be generated *a priori *using the random number function in Excel by an independent investigator not directly involved in assessment of participants. Allocation will be sealed in opaque and consecutively numbered envelopes held in a central location. These will be opened in sequence by a person not involved in the study after recruitment and baseline testing of participants.

### Participants

88 men and women (approximately 30 each of KL grades 2, 3, and 4) aged over 50 years will be recruited from the community via advertisements in local clubs, libraries, and the print and radio media in metropolitan Melbourne, Australia and from our database of people who have registered their interest in participating in research studies. Eligibility will be confirmed by radiographic and clinical examination. People with tibiofemoral joint OA fulfilling American College of Rheumatology classification criteria [31] and reporting average knee pain on walking >3 on an 11-point scale (0 = no pain; 10 = maximal pain) will be included. Other inclusion criteria will be: (i) knee alignment ≤ 182° on a standardised semiflexed standing posteroanterior knee x-ray (which corresponds to a mechanical axis angle of ≤ 180° on a full leg x-ray, indicating varus alignment) [32]; (ii) predominance of pain/tenderness over the medial region of the knee and; (iii) medial compartment radiographic OA defined as at least Grade 1 medial joint space narrowing or Grade 1 medial tibial or femoral osteophytes [33].

Exclusion criteria will include: (i) questionable radiographic knee OA (Kellgren and Lawrence Grade 1 [30]); (ii) lateral tibiofemoral joint space width less than medial; (iii) symptoms originating predominantly from the patellofemoral joint as determined by clinical examination; (iv) knee surgery or intra-articular corticosteroid injection within 6 months; (v) current or past (within 4 weeks) oral corticosteroid use; (vi) systemic arthritic conditions; (vii) history of hip or tibiofemoral/patellofemoral joint replacement or tibial osteotomy; (viii) any other muscular, joint or neurological condition affecting lower limb function; (ix) planning to commence exercise or other treatment for knee OA in the next 12 months; (x) unable to ambulate without a gait aid and; (xi) non-English speaking.

Ethical approval has been obtained from the University of Melbourne Human Research Ethics Committee (HREC No. 0709220) and from the Department of Human Services Victoria, Radiation Safety Committee. All participants will provide written informed consent.

### Interventions

All participants will be requested to refrain from seeking other forms of treatment during the trial. However, due to ethical considerations, analgesia will be permitted as required.

#### Hip strengthening group

Participants will perform a series of 6 exercises designed to strengthen the hip abductor and adductor muscles (Table 1) at home, five times per week for 12 weeks. Strengthening will be limited to the limb with the test knee for 5 of the 6 exercises. In participants with bilateral knee OA, only the most symptomatic knee will be assessed and treated. Participants will attend one of 6 musculoskeletal physiotherapy clinics located around metropolitan Melbourne to receive appropriate instruction of exercises, monitoring, and safe progression of resistance. Participants will visit the physiotherapist on 7 occasions, occurring at 1, 2, 3, 4, 5, 7, and 10 weeks following the baseline assessment. These will be individual sessions lasting 30 minutes initially and 15 minutes for subsequent visits. The exercises will be standardised and the therapists will be trained to deliver the exercises prior to the study. The therapists will adjust the intensity of the exercises accordingly as determined by the participant s ability to complete 10 repetitions for a given exercise.

**Table 1:** Hip abductor and adductor muscle strengthening exercises

**Exercise**	**Dosage**
Abduction in sidelying	3 sets of 10 at a 10 RM resistance
Unilateral hip abduction performed in sidelying with the use of ankle cuff weights	
Abduction in standing	3 sets of 10 with moderate resistance band
Unilateral hip abduction performed in standing with the use of resistance band	
Standing wall isometric hip abduction	3 sets of 10 with 5 second holds
Performed in unipedal stance with the opposite limb in 90 degrees of hip and knee flexion. Exercises will be done for both limbs.	
Adduction in sidelying	3 sets of 10 at a 10 RM resistance
Unilateral hip adduction performed in sidelying with the use of ankle cuff weights if possible	
Abduction in standing	3 sets of 10 with moderate resistance band
Unilateral hip abduction performed in standing with the use of resistance band	
Towel squeezes	3 sets of 10 with 5 second holds
Bilateral isometric hip adduction against a rolled-up towel in sitting	

#### Control group

Control group participants will not receive any additional intervention or complete any home exercises during the 12 week study. They will be requested to continue their usual care.

### Outcome assessment

Participants will be assessed at baseline and at 12 weeks by an assessor blinded to group allocation. Age, gender, duration of knee OA symptoms, previous treatment, surgery and medication use for knee OA will be obtained at the baseline assessment. Radiographic disease severity will be assessed from the baseline x-ray using the Kellgren and Lawrence grading system [30]. Skyline knee x-rays performed in nonweightbearing at 20° knee flexion will also be obtained to assess severity of OA within the patellofemoral joint. X-rays will be evaluated according to osteophytes and joint space narrowing based on a 4 point scale [33].

#### Gait measures

Participants will undergo three-dimensional gait analyses where they will be instructed to walk barefoot at their self-selected normal speed that will be monitored by two photoelectric beams. For the follow-up assessment, participants will again be instructed to walk at their self-selected speed, but will also perform additional trials constrained to the speed exhibited at baseline if the self-selected speed varies by more than 5% between test sessions. Kinematic data will be collected using a Vicon motion analysis system with eight M2 CMOS cameras (1280 × 1024) operating at 120 Hz (Vicon, Oxford, UK). The standard Plug-In-Gait marker set will be used (anterior superior iliac spine, posterior superior iliac spine, mid-lateral thigh, lateral knee joint, lateral shank, lateral malleolus, second metatarsal head, and over the posterior calcaneus). Additional markers will be placed anteriorly at the sternal notch and posteriorly over the spinous processes of T2 and T10 to construct a local segment coordinate system fixed to the thorax. Lastly, medial knee and ankle markers will be included during a static standing trial to determine relative positioning of joint centres. Kinetic data will be collected using a single, floor-mounted 0R6-6-2000 force plate at a sampling rate of 1080 Hz (Advanced Mechanical Technology Inc., Watertown, MA), in synchrony with the cameras. Net joint moments will be calculated via inverse dynamics (Vicon Plug-In-Gait v1.9).

The primary outcome measure will be the peak external knee adduction moment during the stance phase of gait, normalised to body weight and height (Nm/BW*HT %). Test-retest reliability in our laboratory for the knee adduction moment is excellent (intra-class correlation coefficients (ICC(3,5)) of 0.92–0.97, in 11 elderly patients with knee pain tested one week apart). Other variables of interest (see Table 2) will include the peak external hip adduction moment during stance, maximum contralateral pelvic drop, and trunk lean angulation in the frontal plane. A total of five trials will be obtained for the test limb and all data reported will be averaged across the 5 trials at each test session.

**Table 2:** Measures collected in the trial

**Primary Outcome**	**Measurement**
Peak external knee adduction moment	3-D motion analysis and force plate system average of 5 walking trials at self-selected speed

**Secondary Outcomes**	**Measurement**

**Gait Analysis**	
Peak external hip adduction moment	3-D motion analysis and force plate system average of 5 walking trials at self-selected speed
Peak pelvic obliquity and lateral trunk lean angle	3-D motion analysis and force plate system average of 5 walking trials at self-selected speed
	
**Strength**	
Isometric muscle strength of hip abductors, adductors, flexors, and rotators	Hand held dynamometer
Isometric quadriceps muscle strength	KinCom dynamometer
	
**Symptoms**	
Pain, stiffness and physical function in past 48 hours	WOMAC Osteoarthritis Index – Likert version
Average pain over past week in standing, walking and overall	11 point horizontal numeric rating scale (end descriptors of 0 = no pain and 10 = worst pain possible)
Average restriction over past week in activities of daily living	11 point horizontal numeric rating scale (end descriptors of 0 = no restriction and 10 = maximum restriction)
Perceived rating of change for pain and for function over 12 week trial	Ordinal scale (1-much worse, 2-slightly worse, 3-no change, 4-slightly better, 5-much better) at study completion
	
**Function**	
Standing balance	Step test number of times can step foot up and down off 15 cm step in 15 secs
Overall function	Timed stair ascent and descent

**Other Measures**	**Measurement**

Physical activity levels	Physical Activity Scale for the Elderly (PASE)
Compliance (exercise group only)	Number of physiotherapy visits Completion of home exercises via log-book
Adverse effects	Log-book and open probe questioning

#### Strength measures

Participants will undergo isometric strength assessment of hip abduction, adduction, extension, flexion, internal rotation and external rotation as well as knee extension. After a single, submaximal trial for each movement, participants will be instructed to perform 3 trials each of 5 seconds duration, separated by 15 seconds of rest. The maximum force output from the 3 trials will be recorded. All force data will be converted to Nm by multiplying by the resistance lever arm and normalized to body mass (Nm/kg).

Maximal isometric hip abduction and adduction torque will be measured with the participant positioned supine on a height-adjustable examination table, and with adequate stabilization of the pelvis and contralateral limb. The study limb will be positioned in slight abduction with no hip or knee flexion. A handheld dynamometer (HHD) (Nicholas MMT, Lafayette Instruments, Lafayette, IN) will be placed 5 cm proximal to the lateral and medial femoral condyles for the hip abduction and adduction trials, respectively.

Maximum isometric hip extension strength will be measured in the same participant position as used during abduction and adduction testing, except that the test limb will be raised off the bed and supported by a padded cuff [34]. A force transducer connected to an electronic inclinometer will be suspended from the ceiling using a chain, and the transducer-inclinometer device will be attached to the padded cuff. The angle of the chain will be 70 degrees from the horizontal and the involved hip will be flexed 20 degrees to ensure that the test limb is perpendicular to the force transducer. Participants will then perform isometric hip extension while force data are recorded from the force transducer.

Hip strength for flexion, internal rotation, and external rotation will be measured with the participant stabilized securely to a chair and the hips and knees flexed to 90 degrees. The HHD will be placed immediately proximal to the superior pole of the patella for tests of hip flexion strength and immediately proximal to the medial and lateral malleoli for external and internal hip rotation strength, respectively.

Quadriceps isometric muscle strength at 60° of knee flexion will be assessed using the Kin-Com 125-AP dynamometer (Chattecx Corporation, Chattanooga, TN, USA). Participants will be seated in a standardised position and stabilized to the testing apparatus with straps as well as a thigh block. The lever arm axis of rotation will be aligned with the lateral femoral condyle for each patient.

All of the above strength tests have demonstrated excellent test-retest reliability in our lab (ICC_2,2 _= 0.84–0.98) based on a subgroup of patients who were tested on 2 separate occasions separated by an average of 14 days (range 12–23 days).

#### Self-report measures

Overall average knee pain in the past week will be self-assessed by a single, 11-point horizontal numeric rating scale with terminal descriptors of (0 = no pain; 10 = maximal pain). Similar scales will be used to measure average pain in the past week for each of walking and standing. Such measurement has demonstrated reliability in patients with knee OA [35].

Self-reported knee pain, stiffness and difficulty with physical function will be measured using the Western Ontario and McMaster Universities (WOMAC) Osteoarthritis Index [36], a valid, reliable, and responsive disease-specific instrument. The WOMAC consists of 24 questions covering pain (scored 0–20), stiffness (scored 0–8) and physical function (scored 0–68), where higher scores indicate worse symptoms.

Participants will rate their perceived change in pain and in physical function over the 12 weeks (compared to baseline) on an ordinal scale (1-much worse, 2-slightly worse, 3-no change, 4-slightly better, 5-much better). Scales of this kind are frequently used as an external criterion for comparison with changes in scores of other outcomes [37]. Measuring patient perceived improvement using a rating of change scale has been shown to be a clinically relevant and stable concept for interpreting truly meaningful improvements from the individual perspective [38].

#### Objective measures of function

Observed functional tests will include the step test and a timed stair ascent/descent task. The step test is a functional, dynamic test of standing balance [39]. Participants will stand barefoot on the study leg in front of a 15 cm step and will be instructed to step the opposite foot on and off the step as quickly as possible over 15 seconds. The number of times the foot can be placed up onto the step and returned to the floor is recorded, with higher scores indicating better balance. The stair ascent/descent task involves ascending and descending a set of stairs with six steps (each 17.5 centimetres in height). Participants will be instructed to climb up, turn around at the top of the stairs and climb down at their usual pace and the total time taken will be recorded, with longer time taken indicating poorer physical function.

#### Other measures

Physical activity will be measured using the Physical Activity Scale for the Elderly (PASE) which assesses both the level and type of recreational and occupational physical activity undertaken by participants over the previous week. The PASE was developed and has been validated in samples of older adults (age 55+ years) [40]. Scores on the PASE range from 0 to over 400, and higher scores indicate higher activity levels.

In addition to adverse effects over the course of the trial, use of medications and other therapies will be recorded by each participant in a personal logbook.

### Sample size calculations

Our sample size of 88 ensures adequate power to detect significant changes in the peak knee adduction moment after 12 weeks. We estimate a change in the peak knee adduction moment after a strengthening program to be between 5 and 10%. Based on our participants with knee OA from previous studies, a 7.5% reduction equates to an absolute change of approximately 0.3 Nm/BW*HT %. Estimates of the standard deviation of change scores are 0.4 Nm/BW*HT % based on data obtained from ongoing studies in our laboratory investigating conservative treatment for knee OA as well as other published reports [41]. Thus, a total of 38 patients per group are required to detect a 0.3 Nm/BW*ht % change with 90% power (2 sided test, alpha = 0.05). We also assume an attrition rate of approximately 15% for the duration of the study, suggesting that 44 patients per group will be required.

### Statistical analysis

Data will be assessed in a blinded fashion. All analyses will be conducted on an intention-to-treat principle using all randomised participants. Missing data will be replaced by the last score carried forward. Demographic characteristics and baseline data will be summarised by descriptive statistics. For outcomes measured on a continuous scale, differences in mean change from baseline to 12 weeks between groups will be evaluated using linear regression modelling while adjusting for baseline levels of the outcome measure. For the knee adduction moment, gait speed, disease severity (KL grade), and pain will also be included as covariates. Model assumptions will be checked by standard diagnostic plots [42]. Participant rating of perceived change will be compared between the two groups by calculating the relative risks and their 95% confidence intervals using log binomial regression [43].

## Discussion

This study uses a single-blind randomised controlled trial design to investigate whether strengthening of the hip abductor and adductor muscles for 12 weeks in people with medial knee OA and varus malalignment reduces the knee adduction moment during walking gait. As a secondary aim, it will evaluate whether hip muscle strengthening also improves disease-associated symptoms, namely knee pain and physical function.

Lower limb muscle strengthening has recently received increased interest as an inexpensive treatment for knee OA due to its ability to reduce knee pain and improve physical function [23], and also because of its potential ability to reduce knee joint load. The novelty of the present study is the focus on hip strengthening. The hip muscles, particularly the abductors, play an important role in stablization of the pelvis and trunk. Indeed, movement of the contralateral pelvis or lateral leaning of the trunk over the stance limb, which may occur as a result of hip muscle weakness, has been suggested to adversely influence the magnitude of the knee adduction moment [26]. Thus, hip muscle activity appears to be an important, yet understudied, contributor to knee joint load.

We have chosen a series of six exercises to strengthen the hip abductors and adductors based on ease of performance and isolation of the targeted musculature. We have purposely avoided any exercises that involve components of internal or external rotation or that require muscles other than the abductors or adductors as the prime mover. It is acknowledged that human gait requires movement and stabilization in all three planes of movement, but we have focused on only the frontal plane given the established importance of kinematics and kinetics in this plane on knee OA pathophysiology [13,44].

Some authors propose that gait biomechanics in people with knee OA may be influenced by KL grade [45], suggesting a possible confounding influence of disease severity on the effects of interventions in this patient population. Thus, we will recruit equal numbers of participants with mild (KL grade 2), moderate (KL grade 3), and severe knee OA (KL grade 4) and perform post hoc exploratory subgroup analyses. Indeed, a potential benefit of our design is the identification of groups of patients in which conservative treatment such as hip muscle strengthening may or may not be beneficial.

The peak external knee adduction moment was chosen as the primary outcome measure in this study due to its association with medial compartment knee joint load and knee OA progression [11,15]. Thus, it represents a non-invasive, functional means to quantify knee joint loading and serves as a biomarker for disease progression [46]. Given the design of the study, measurement of muscle strength also constitutes an important outcome measure. To ensure that strength gains are limited to the hip abductors and adductors, measurement of hip flexion, extension, rotation (both internal and external), and knee extension will also be conducted. Isometric muscle strength measured with isokinetic and handheld dynamometry have both been shown to be valid and reliable in a clinical setting [47]. Lastly, objective and self-report measures of function, as well as self-reported measurement of pain will provide clinically relevant estimates of change in knee OA symptoms with our novel hip strengthening intervention. Therefore, this study will quantify the effects of hip strengthening on biomechanical, functional, and clinical attributes of knee OA.

It is anticipated that testing of all participants will be completed by the end of 2008. The results from this trial will contribute to evidence based recommendations for the usefulness of hip muscle strengthening in the management of medial knee OA.

## Competing interests

The author(s) declare that they have no competing interests.

## Authors' contributions

KB, DH and RH conceived and designed the trial protocol. KB, DH, RH and  TW procured the project funding. MH is the research assistant on the  project. KB and MH drafted the manuscript and RH and TW contributed to  the manuscript. All authors read and approved the final manuscript.

## Pre-publication history

The pre-publication history for this paper can be accessed here:


